# Severe hyponatremia with consciousness disturbance after receiving SARS-CoV-2 mRNA vaccination

**DOI:** 10.1530/EDM-23-0004

**Published:** 2023-07-17

**Authors:** Takuya Kumagai, Syohei Koyama, Haruka Yorozu, Ayaka Kokita, Naoko Shimizu, Yumi Suganuma, Takashi Goto

**Affiliations:** 1 Postgraduate Clinical Training Center, Akita Red Cross Hospital, Akita, Japan; 2 Department of Metabolism, Akita Red Cross Hospital, Akita, Japan; 3 Department of Gastroenterology, Akita Red Cross Hospital, Akita, Japan

**Keywords:** Adult, Female, Asian - Japanese, Japan, Pituitary, Pituitary, Unique/unexpected symptoms or presentations of a disease, July, 2023

## Abstract

**Summary:**

There are very few reports of syndrome of inappropriate antidiuresis hormone secretion (SIADH) after receiving severe acute respiratory syndrome coronavirus 2 (SARS-CoV-2) mRNA vaccine. Herein, we present the case of an 84-year-old woman who developed severe hyponatremia following the second administration of the SARS-CoV-2 mRNA vaccine. The patient presented with nausea, vomiting, and headache. Laboratory tests showed a plasma sodium level of 119 mmol/L. After receiving 500 mL of intravenous saline over a 2-h period, her plasma sodium level raised to 121 mmol/L, but her symptoms persisted. Considering that rapid plasma sodium correction was necessary, we started 3% saline solution overnight. Her plasma sodium level raised to 132 mmol/L and her symptoms completely resolved. Clinical and laboratory findings were consistent with a diagnosis of SIADH. In the absence of any other triggering factors, we concluded that the condition was likely associated with the vaccination. Clinicians should be aware of the potential for hyponatremia, particularly SIADH, associated with SARS-CoV-2 mRNA vaccination.

**Learning points:**

- Reports of syndrome of inappropriate antidiuresis hormone secretion after receiving severe acute respiratory syndrome coronavirus 2 (SARS-CoV-2) vaccination are limited.
- If nausea, headache, and confusion are observed immediately after SARS-CoV-2 vaccination, clinicians should consider the presence of hyponatremia.
- As similar case reports to date have presented with severe hyponatremia, prompt treatment may be required.

## Background

Severe hyponatremia, defined by serum sodium level below 120 mmol/L, is a potentially lethal condition that can lead to neurological complications. While there have been several reported cases of severe hyponatremia due to syndrome of inappropriate antidiuretic hormone secretion (SIADH) occurring after severe acute respiratory syndrome coronavirus 2 (SARS-CoV-2) vaccination, the relationship between vaccination and SIADH remains unclear. We present a case of severe hyponatremia following SARS-CoV-2 mRNA vaccination that improved with the administration of a 3% saline solution.

## Case presentation

An 84-year-old woman presented to our department with nausea, vomiting, and headache after receiving the second dose of the Pfizer-BioNTech SARS-CoV-2 mRNA vaccine two days before. The first dose, also from Pfizer-BioNTech, was administered three weeks prior to the second dose. Her symptoms gradually worsened, leading to lethargy and impaired responsiveness to questions and commands. Physical examination revealed a body temperature of 36.8°C, blood pressure of 143/75 mmHg, pulse rate of 93 beats per minute, respiratory rate of 20 per minute, and oxygen saturation of 98% on ambient air. She was lethargic and did not respond to questions or commands well. Her mouth was slightly dry but otherwise unremarkable. Her comorbidities were hypertension and atrial fibrillation. She had no significant medical history. Her medications were candesartan 4 mg and apixaban 5 mg; no diuretics were included.

## Investigation

Laboratory tests revealed a plasma sodium level of 119 mmol/L, chloride level of 84 mmol/L, and plasma osmolality of 253 mOsm/L. Urine sodium level was 69.6 mmol/L and urine osmolality was 439 mOsm/L. Results of liver biochemistry, renal function, basal cortisol, and thyroid function were within the normal range (Table 1). Chest x-ray and brain CT scan were unremarkable.

**Table 1:** Laboratory findings on admission.

Investigations	Result	Reference range
Blood cell count		
WBC, /μL	6000	4000–6000
RBC, /μL	353 × 10^4^	450–510
Hb, g/dL	11.6	12.0–16.0
Ht, %	32.9	39.0–51.0
Platelet, ×10^4^/μL	20.4	15.0–41.0
Biochemistry		
Na, mmol/L	119	136–145
K, mmol/L	3.8	3.6–5.1
Cl, mmol/L	84	98–108
Glucose, mg/dL	145	83–110
Cr, mg/dL	0.72	0.61–1.08
BUN, mg/dL	15.7	8.0–22.0
UA, mg/dL	4.4	3.0–7.0
Osmolality, mOsm/L	253	270–295
CRP, mg/dL	0.21	<0.3
Urinary biochemistry		
Na, mmol/L	69.6	
K, mmol/L	38.0	
Cl, mmol/L	75.8	
Cr, mg/dL	69.6	
Osmolality, mOsm/L	439	40–1300
Hormonal data		
ACTH, pg/mL	30.5	7.0–56.0
Cortisol, μg/dL	15.4	5.6–29.7
TSH, μIU/mL	1.53	0.35–4.94
FT3, pg/mL	2.15	2.2–4.3
FT4, ng/dL	1.7	0.9–1.7
AVP, pg/mL	1.1	0.3–4.2

ACTH, adrenocorticotropic hormone; AVP, arginine vasopressin; BUN, blood urea nitrogen; Ca, calcium; Cl, chloride; Cr, creatinine; FT3, free triiodothyronine; FT4, free thyroxine; Hb, hemoglobin; K, potassium; Na, sodium; RBC, red blood cell; TSH, thyrotropin; UA, uric acid; WBC, white blood cell.

## Treatment

**Figure 1:**
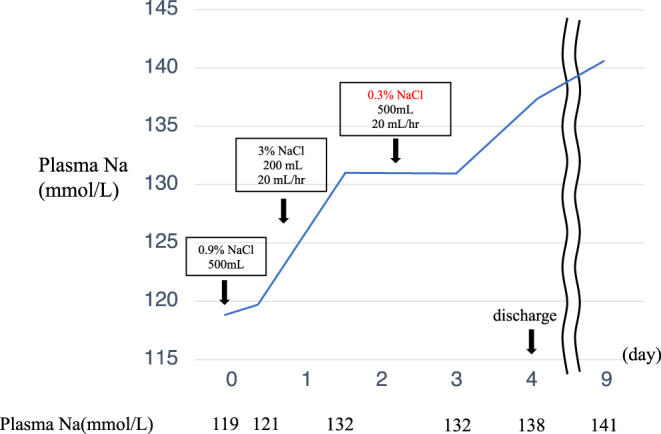
Changes in plasma sodium level and infusion during the clinical course. Clinical course: The patient initially presented with plasma Na of 119 mmol/L. After receiving 500 mL saline, plasma Na was raised to 121 mmol/L. As symptoms persisted, the patient received 200 mL 3% NaCl. The following day, the patient was symptom free and her plasma sodium level raised to 132 mmol/L. The infusion was then changed to 0.3% NaCl and terminated after 24 h of administration. The sodium level was confirmed to be normalized and the patient was discharged.

Medical records revealed that the patient had a normal plasma sodium level in the previous year (Na: 140 mmol/L). The patient was diagnosed with acute symptomatic hyponatremia and was initiated 500 mL of intravenous saline to exclude decreased extracellular fluid volume. Subsequently, her plasma sodium level slightly increased to 121 mmol/L. At this point, her plasma AVP level was 1.1 pg/mL and her plasma osmolality was 268 mOsm/L, compatible with SIADH. As her symptoms persisted, we changed to a 3% saline solution at a rate of 20 mL/h overnight. The following day, the patient was symptom free and her plasma sodium level raised to 132 mmol/L. We changed the fluid to 0.3% NaCl (Na+: 50 mEq/L, K+: 20 mEq/L, Cl−: 50 mEq/L) to avoid osmotic demyelination syndrome because the correction rate of sodium was faster than expected. On hospital day 4, the patient was free from fluid therapy and was discharged with a plasma sodium level of 138 mmol/L (Fig. 1).

## Outcome and follow-up

As there were no other triggering factors apart from vaccination, we considered that SIADH was associated with previous vaccination. Five days after discharge, her plasma sodium level was 141 mmol/L, and symptoms have not recurred to date. She continues to visit the primary care clinic.

## Discussion

Various adverse events have been reported after receiving SARS-CoV-2 mRNA vaccination. Reports ranged from common symptoms such as fever, headache, and fatigue to severe diseases such as thrombosis with thrombocytopenia syndrome (1).

To date, several cases of SIADH associated with coronavirus disease 2019 have been reported, and the mechanism is being elucidated (2). On the other hand, reports of SIADH associated with vaccination are very few (3, 4, 5, 6, 7). We experienced the case of severe hyponatremia after receiving SARS-CoV-2 mRNA vaccination. Upon admission, while there were no overt signs of dehydration such as tachycardia or hypotension, the possibility of mild dehydration due to vomiting and poor oral intake could not be excluded and extracellular fluid was administered. Despite this, the hyponatremia and its associated symptoms did not improve with initial intravenous infusion. As the results of the laboratory examination were consistent with SIADH, we concluded that SIADH was the most likely diagnosis based on the clinical course and laboratory findings. There were no signs of inflammation, pulmonary lesions, or other factors causing SIADH, and the time course suggested that SIADH could be related to vaccination. In previous case reports similar to the present case, it has been postulated that the increase in inflammatory cytokines such as C-reactive protein (CRP) and interleukin (IL)-6 induced by vaccination stimulates antidiuretic hormone secretion (7). Furthermore, another study in mice showed that SARS-CoV-2 mRNA vaccination induces escalation of plasma IL-6 levels (8). Although IL-6 levels were not measured in this case, a slight elevation of CRP was observed. It is possible that the increase in inflammatory cytokines due to vaccination may be associated.

Another possible diagnosis in this case is mineralocorticoid-responsive hyponatremia of the elderly (MRHE), a syndrome first described by Ishikawa *et al.* in 1987 as a differential diagnosis of SIADH, particularly among elderly patients (9). This syndrome is characterized by age-related decreased sodium reabsorption and hyporesponsiveness of the renin–angiotensin–aldosterone system. As the patient in this case was elderly and taking an angiotensin receptor blocker, she was considered to be at high risk for MRHE. However, despite the fact that MRHE typically requires discontinuation of angiotensin receptor blockers and supplementation with mineralocorticoids to improve plasma sodium levels (10), improvement was achieved in this case without such management, leading us to conclude that SIADH was the more likely diagnosis.

In this case, the patient presented with severe hyponatremia and impaired consciousness, a potentially life-threatening condition, that improved with prompt administration of 3% saline solution. Hyponatremia should be considered in the differential diagnosis of patients presenting with unexplained nausea, headache, or disorientation after vaccination, as severe hyponatremia can occur and should not be overlooked. It is possible that cases of hyponatremia following vaccination may be more common than reported, as some patients with mild symptoms may not seek medical evaluation. Clinicians should be aware of the potential for hyponatremia, particularly SIADH, associated with SARS-CoV-2 mRNA vaccination.

The primary limitation of this case report is that the cause of SIADH has only been presumptively linked to the SARS-CoV-2 mRNA vaccination and has not been definitively proven. Additionally, the exclusion of MRHE as a diagnosis is uncertain as renin and aldosterone levels were not measured. Further research is needed to clarify the pathophysiology of SIADH potentially caused by SARS-CoV-2 mRNA vaccination.

## Declaration of interest

The authors declare that there is no conflict of interest that could be perceived as prejudicing the impartiality of the research reported.

## Funding

This study did not receive any specific grant from any funding agency in the public, commercial, or not-for-profit sector.

## Patient consent

Written informed consent for publication of their clinical details and clinical images was obtained from the patient.

## Author contribution statement

All authors participated in the treatment of the patient, collected data, interpreted data, and wrote the manuscript. All authors read and approved the final manuscript.

## References

[1] See I SU JR Late A Woo EJ See I Su JR Lale A Woo EJ Guh AY Shimabukuro TT US case reports of cerebral venous sinus thrombosis with thrombocytopenia after Ad26.COV2.S Vaccination, March 2 to April 21, 2021. JAMA 2021 325 2448–2456. (10.1001/jama.2021.7517) 33929487 PMC8087975

[2] Yousaf Z Al-Shokri SD Al-soub H & Mohamed MFH. COVID-19-associated SIADH: a clue in the times of pandemic! American Journal of Physiology-Endocrinology and Metabolism 2020 318 E882–E885. (10.1152/ajpendo.00178.2020) 32396497 PMC7276977

[3] Lindner G & Ryser B. The syndrome of inappropriate antidiuresis after vaccination against COVID-19: case report. BMC Infectious Diseases 2021 21 1000. (10.1186/s12879-021-06690-8) PMC8464133 34560836

[4] Chienwichai K Sriinkua P & Chang A. Symptomatic hyponatremia after ChAdOx1 nCoV-19 coronavirus disease-19 vaccination. Clinical Nephrology 2022 98 162–166. (10.5414/CN110906) 35818815

[5] Jud P Hackl G Reisinger AC Horvath A Eller P & Stadlbauer V. Red urine and a red herring - diagnosing rare diseases in the light of the COVID-19 pandemic. Zeitschrift für Gastroenterologie 2022 60 1326–1331. (10.1055/a-1659-4481) 34768287 PMC9470277

[6] Desra A Smith J & Chiang C. A case of post-covid vaccination hyponatraemia. Pathology 2022 54(Supplement4). (10.1016/j.pathol.2021.12.015)

[7] Yang JW Kim YR & Yu Ah H. Syndrome of inappropriate antidiuretic hormone and status epilepticus associated with mRNA-based SARS-CoV-2 vaccination. International Urology and Nephrology 2022 30 1–2. 10.1007/s11255-022-03451-7 PMC9800232 36581709

[8] Gebre MS Rauch S Roth N Gergen J Yu J Liu X Cole AC Mueller SO Petsch B & Barouch DH. mRNA vaccines induce rapid antibody responses in mice. npj Vaccines 2022 7 88. (10.1038/s41541-022-00511-y) PMC9340693 35915094

[9] Ishikawa SE Saito T Kaneko K Okada K & Kuzuya T. Hyponatremia responsive to fludrocortisone acetate in elderly patients after head injury. Annals of Internal Medicine 1987 106 187–191. (10.7326/0003-4819-106-2-187) 3800181

[10] Sogabe T Ishida K Nakakura H Noborio M & Kinoshita Y. Mineralocorticoid-responsive hyponatremia of the elderly secondary to aspiration pneumonia: a case report. Journal of Japanese Association for Acute Medicine 2019 30 189–219.

